# The developmental English Lexicon Project (d-ELP): Continuous word reading difficulty estimates of the 9,961 most frequently printed English words for US children

**DOI:** 10.3758/s13428-026-03222-7

**Published:** 2026-11-04

**Authors:** Donald L. Compton, Jay G. Rueckl, Laura M. Steacy, Noam Siegelman, Ashley A. Edwards, Yaacov Petscher, Nicole Patton-Terry, Matthew J. Cooper Borkenhagen, Chelsea Starke

**Affiliations:** 1 Florida Center for Reading Research, Tallahassee, FL USA; 2 Australian Centre for the Advancement of Literacy, North Sydney, Australia; 3 University of Connecticut, Storrs, CT USA; 4 Hebrew University, Jerusalem, Israel

**Keywords:** Word reading development, Word difficulty, d-ELP

## Abstract

The developmental English Lexicon Project (d-ELP) is a database comprising word reading data for 9,961 of the most frequently printed English words for children, including continuous item response theory (IRT)-based estimates of word reading difficulty, accompanying word characteristics, and raw trial-level data. Data on isolated word reading accuracy were collected from 1,907 children in grades 1–5 across multiple US sites. Each child read a list of words ranging from 250 to 500 items depending on reading skill (less proficient children were often unable to finish the entire list of words), with each list representing the full distribution of word difficulty oversampled for words at the child’s reading level. Each child also attempted 99 linking items representing the full distribution of word difficulty. A total of 631,322 responses were collected, with the average child providing 336. For the final sample of words, the average number of responses per word for the 9,862 non-linking items was 47.14 (range of 10–592), and for the 99 linking items the average was 1,681.42 (range of 1,083–1,907). A Rasch model, with planned missing data design, was fit to children’s responses on the word list and linking item responses. This introduction describes the motivation for this project, the methods used for data collection, the analytic framework used to establish a continuous measure of word reading difficulty across words, descriptive statistics of the relational database and initial behavioral results, and a publicly available search engine providing searchable access to word difficulty ratings and associated word-level statistics.

The English orthography exhibits probabilistic, rather than strictly rule-governed, mappings between orthography and phonology across multiple grain sizes (see Ziegler & Goswami, 2005; Venezky, 1999). Its structure reflects a complex interplay of morphological regularities, syllabic constraints, and numerous sublexical regularities in spelling–sound correspondences—features that have led Seidenberg (2005, 2017) and colleagues (e.g., Plaut et al., 1996; Rueckl & Seidenberg, 2011; Seidenberg et al., 2020; Seidenberg & McClelland, 1989) to characterize English as *quasiregular*. For this reason, English poses unique and significant challenges for children as they work to develop accurate and efficient word reading skills (see Seymour, 2013; Seymour et al., 2003). Consequently, the English orthography gives rise to substantial individual differences in word reading accuracy in developing readers, reflecting the joint effects of various word-level characteristics such as frequency, length, and orthography-to-phonology and orthography-to-semantics consistency (for details, see Castles et al., 2018; Compton et al., 2014; Deacon & Levesque, 2024; Kearns, 2015, 2020; Kearns et al., 2016; Leslie & Calhoon, 1995; Martens & de Jong, 2006; Marinus et al., 2015; Rueckl, 2016; Seidenberg, 1989; Siegelman et al., 2020a, 2020b; Steacy & Compton, 2019; Steacy et al., 2022; Treiman et al., 1990; Wade-Woolley & Heggie, 2015).

Early work by Seidenberg and colleagues (e.g., Backman et al., 1984; Seidenberg et al., 1984; Waters et al., 1984) and subsequent studies examining children’s word naming and word identification established that variables such as word frequency, spelling–sound consistency, word length, and neighborhood structure influence reading performance, with effects often varying as a function of reading skill. These findings helped establish the view that word reading is shaped not only by characteristics of the reader but also by statistical regularities of words. Subsequent studies extended this work by examining increasingly large sets of words and a broader range of lexical characteristics. For example, Spencer (2010) investigated the effects of frequency, transparency, and other lexical variables on the reading accuracy of British 6-year-olds across a set of approximately 150 words. This study demonstrated that substantial variation in children’s word reading accuracy could be explained by characteristics of individual words. More recent investigations have expanded the range of lexical variables examined, incorporating semantic characteristics (e.g., imageability, concreteness, and age of acquisition), morphological structure, orthographic and phonological neighborhood measures, and indices of spelling–sound consistency (e.g., Apfelbaum et al., 2013; Kearns et al., 2016; Steacy et al., 2019; Kellenberger et al., 2024; Yap et al., 2012). Collectively, this work has shown that word-level characteristics account for meaningful variation in children’s word reading performance and that the influence of these characteristics often interacts with child-level factors such as decoding skill, reading experience, and vocabulary knowledge.

Despite these advances, most developmental studies have been limited by relatively small word samples, often ranging from a few dozen to a few hundred words. As a result, estimates of the influence of lexical characteristics may be constrained by the particular words selected for study. Large-scale item-level databases such as the developmental English Lexicon Project (d-ELP) provide an opportunity to examine lexical influences across thousands of words spanning a broad range of difficulty levels and lexical characteristics. Such databases can support more comprehensive investigations of how established lexical characteristics—including frequency, length, transparency, semantic variables, and neighborhood structure—contribute to word reading difficulty in developing readers and provide a foundation for evaluating the contribution of emerging theoretically motivated predictors.

Attempts to explore, understand, and predict the combined effects of multiple word-level characteristics on word reading accuracy in developing readers have been constrained by several limitations in the developmental reading literature, as follows: (1) although the field has produced several large-scale databases of word recognition—commonly referred to as “lexicon projects” and related “crowdsourcing projects” (e.g., Adelman et al., 2014; Balota et al., 2007; Keuleers et al., 2012; Mandera et al., 2020)—these resources primarily reflect adult reading behavior; (2) empirical studies of word reading acquisition have predominantly focused on monosyllabic, monomorphemic words (e.g., Balota et al., 2007; Coltheart et al., 2001; Perry et al., 2010; Roberts et al., 2011; Seidenberg & McClelland, 1989; Siegelman et al., 2020a, 2020b; Yap & Balota, 2009); (3) most developmental studies rely on small samples of both children and words and employ factorial designs with limited control variables (e.g., Strain et al., 2002); and (4) publicly available developmental databases generally do not provide word difficulty estimates linked to the lexical, orthographic, and functional characteristics of words commonly encountered in children’s print input, including both published (e.g., Korochkina & Rastle, 2025) and unpublished (e.g., Dawson et al., 2023) sources.

To address these limitations, we developed the developmental English Lexicon Project (d-ELP), a publicly available relational database containing continuous item response theory (IRT)-based estimates of oral word reading difficulty for 9,961 high-frequency English words encountered by US children in grades 1–5, together with a broad set of accompanying word characteristics. The database was designed to support detailed modeling of the lexical and orthographic properties that contribute to word reading difficulty across a large number of items and a relatively diverse sample of English-speaking children. One notable exception is the Developmental Lexicon Project (DLP; Schröter & Schroeder, 2017), which collected lexical decision and naming data from German-speaking children on 1,152 words and provided an important developmental counterpart to adult lexicon projects. The d-ELP extends this work in several important respects. First, rather than reporting performance on a fixed set of items, the d-ELP uses an item response theory (Rasch) framework within a planned-missing-data design, with a common set of linking items used to place word difficulty estimates on a shared developmental scale. This approach permits direct comparison of difficulty estimates across nearly 10,000 words even though individual children completed only a subset of the full item pool. Second, the d-ELP substantially expands the lexical coverage of developmental databases. Third, whereas the DLP was developed for German, a relatively transparent orthography, the d-ELP focuses on English, whose quasiregular orthography presents unique challenges for developing readers. Finally, whereas the DLP examined lexical decision and naming performance, the d-ELP was designed to estimate oral word reading^1^ difficulty, a construct that is directly aligned with developmental theories of English word reading acquisition and with educational assessment and instructional practice.

Beyond providing developmental word difficulty estimates, the d-ELP can support validation of age-appropriate psycholinguistic variables, selection of item subsets for “virtual experiments” (e.g., Kuperman, 2015), and construction of item sets for developmental, experimental, and individual-differences studies. More broadly, the database provides developmental data for evaluating and refining computational models of word reading development; constructing instructional materials appropriate for developing readers and spellers, including artificial intelligence (AI)-based assessment and instructional systems; and developing more precise, developmentally appropriate formative and summative assessments of word reading skill. The project was also motivated by continuing questions about the magnitude of effects associated with specific word-level variables and about which variables should be incorporated as controls in factorial experiments and individual-differences studies (see Balota & Cortese, 2012; Brysbaert & Mandera, 2014; Keuleers & Balota, 2015).

In this introduction to the d-ELP, we describe the motivation for this project, the methods used for data collection, the analytic framework used to establish a continuous measure of word reading difficulty across a wide swath of printed words, descriptive statistics of the relational database, and an overview of the functionality of the publicly available relational database that provides searchable access to the oral word difficulty ratings and associated word-level statistics for each of the stimuli (along with the raw trial-level data). We also demonstrate the d-ELP’s ability to estimate the impact of general word-level predictors (e.g., frequency, word length, transparency, semantic measures, and orthographic neighborhood size) on word recognition performance in developing readers to help demonstrate the potential of the database to inform mechanisms associated with early word reading development and identify potentially important word characteristics to be included in smaller-scale experiments of word reading performance in developing readers.

It is important to note that the word difficulty estimates provided by the d-ELP reflect difficulty in oral word reading rather than a broader construct of lexical knowledge. Although oral word reading is strongly influenced by orthographic, phonological, semantic, and morphological characteristics, successful performance on a word naming task does not necessarily imply complete knowledge of a word’s meaning, nor does knowledge of a word’s meaning guarantee accurate pronunciation. Consequently, the d-ELP should be interpreted as providing item-level estimates of oral word reading difficulty, a construct that is highly relevant to developmental theories of reading acquisition as well as educational assessment and intervention. For simplicity we use “word difficulty” throughout the manuscript to refer to “oral word reading difficulty in developing readers.”

## Predicting English oral word reading difficulty in developing readers

Children’s word reading development follows a curvilinear growth pattern, with more rapid progress in early childhood and slower growth as they get older (for details see Francis et al., 1996). By eighth grade, a typically developing reader can recognize roughly 10,000 unique word forms, excluding inflectional variants (Ehri, 2005; Harris & Jacobson, 1982). The development of word reading skill in children has traditionally been characterized at a micro level by item-learning processes (e.g., Share, 1995) and at a macro level by stage-based processing (Ehri, 2020). Item learning refers to the process by which children gradually establish high-quality lexical representations through phonological recoding and repeated encounters with print. Through the process of fully analyzing each word, typically through phonological recoding, a unique orthographic entry is added to the lexicon, enabling efficient, automatic retrieval and access to phonological, syntactic, and semantic information during reading (Castles et al., 2018; Humphreys, 1985; Perfetti, 1992; Perfetti & Hart, 2001; Perfetti & Stafura, 2014; Snow, 2002; Stanovich, 1991). Stage-based theories, in contrast, describe developmental regularities in the strategies and knowledge, both implicit and explicit, that children bring to word identification. These models specify how a child’s available linguistic knowledge—phonological, orthographic, and semantic—conditions the probability of successfully reading a given word, thereby linking child-level skill with word-level properties. Early stage-based models characterized this evolution as a series of qualitative shifts from visual recognition to phonological decoding and ultimately to automatic word recognition (e.g., Chall, 1983; Ehri, 1995; Frith, 1985). Modern perspectives increasingly view these stages not as discrete but as emergent from continuous item-level learning. Children’s experience-driven tuning of orthography–phonology and orthography–semantics mappings lead to the apparent stage-like patterns observed at the behavioral level, while promoting expanded and strengthened connections between orthography, phonology, and semantics (Siegelman, Kearns, et al., 2020). This evolving lexical organization enables children to generalize learned patterns to increasingly varied and challenging forms, ultimately allowing them to process novel and ever more complex letter strings with greater flexibility and efficiency (see Share, 2025; Siegelman, Rueckl, et al., 2020).

## Child-by-word models of word reading development

The emergence of explanatory item response models (see De Boeck & Wilson, 2004) has allowed the merging of micro- and macro-perspectives into what is now referred to as “child-by-word” models of word reading development (e.g., Harm & Seidenberg, 2004; Nation & Snowling, 1988; Steacy et al., 2022). Child-by-word models of word reading development assert the following: word reading is not uniform across words or readers; the same child may read different words with varying success depending on word properties; the same word may be read differently by different children depending on a child’s reading skills and experience; and thus, reading development involves interactions between the attributes of the word and characteristics of the child, including experiences with text (e.g., Kellenberger et al., 2024; Petscher et al., 2020; Steacy, 2020). These child-by-word differences are shaped, in large part, by children’s statistical learning of orthography–phonology and orthography–semantic correspondences (see Siegelman, Rueckl, et al., 2020). Through repeated exposure to print, statistical learning mechanisms allow children to detect and internalize the probabilistic relationships between written and spoken word forms, becoming increasingly sensitive to the covariance structure linking orthographic patterns with their phonological and semantic representations in English (e.g., Apfelbaum et al., 2013; Chetail, 2015; Nation & Castles, 2017; Treiman & Kessler, 2006). This growing sensitivity supports the development of a probabilistic mapping system that facilitates decoding and word recognition, enabling readers to navigate the quasiregular nature of the English orthography—capitalizing on high-frequency patterns while flexibly adapting to less common mappings (Sawi & Rueckl, 2019; Steacy et al., 2019; Treiman et al., 2003, 2006).

**Fig. 1:**
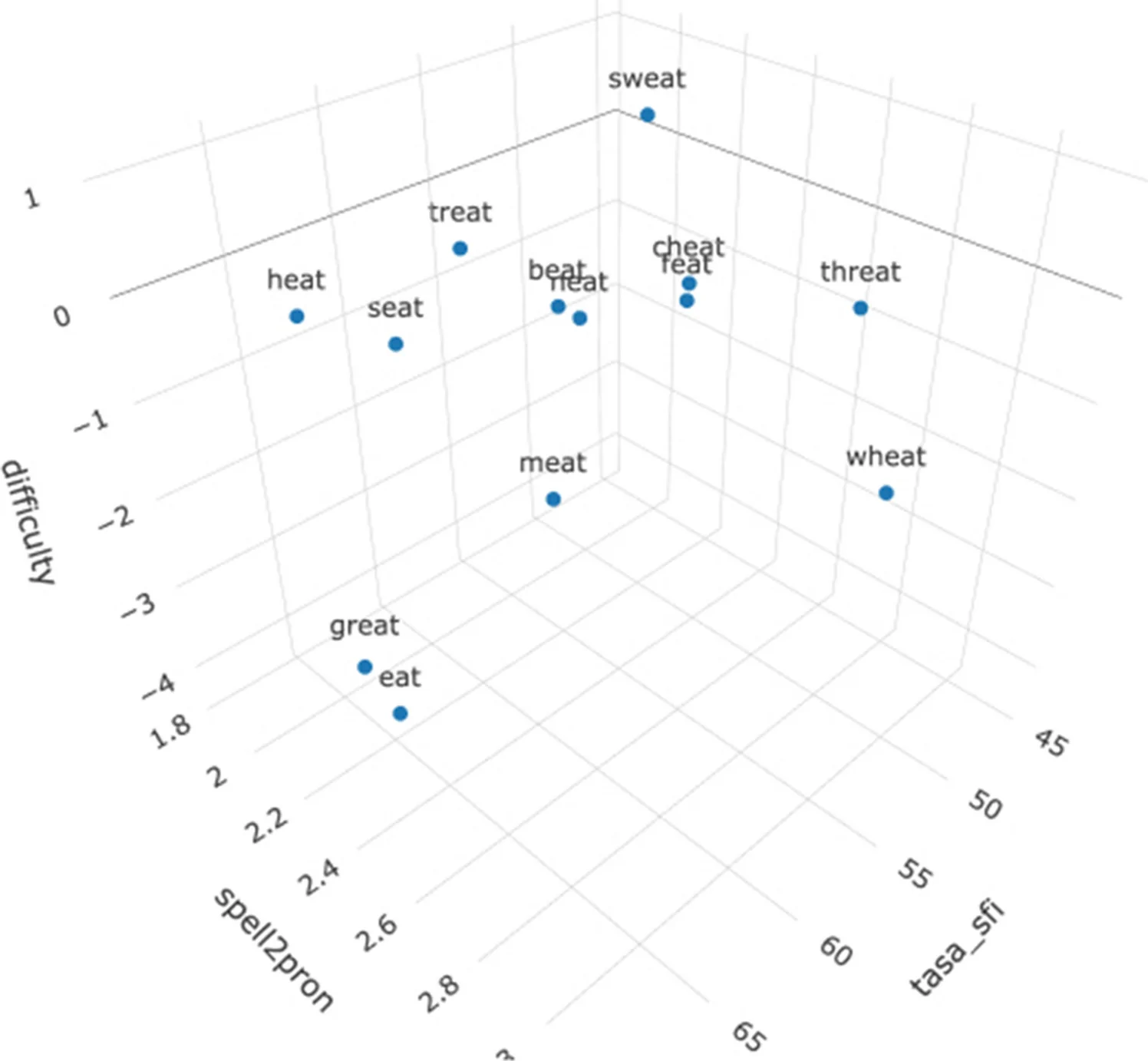
Word difficulty (difficulty) as a function of frequency (tasa_sfi) and regularity (spell2pron) for single-syllable words with the -eat rime pattern. *Note.* tasa_sfi= standard frequency index (Zeno et al., 1995); spell2pron = spelling-to-pronunciation transparency (Edwards et al., 2024)

To illustrate, early connections between orthography and phonology in developing readers are initially based on simple grapheme-phoneme correspondences that are relatively insensitive to orthographic context (see de Jong & Molenaar, 2025; Share, 1995). With reading development, initially incomplete and oversimplified representations evolve to become more sophisticated context-dependent connections (see Steacy et al., 2019; Treiman et al., 2003, 2006). A good example of this is the *ea* diagraph which appears frequently in one-syllable words and is inconsistent in its pronunciation. As children develop word reading skill and practice reading in text, they encounter various pronunciations of *ea*, such as/i/in *meat*,/ɛ/in *head*, and/eɪ/in *great*, along with other less frequent pronunciations (e.g.,/ə/in *ocean* and/i/in *Guinea*). Experiences with “*ea*-words” shape and influence the nature of orthography-to-phonological connection for *ea*, slowly evolving into a set of representations that mirror the probabilistic relationship between orthography and phonology for *ea* in the English corpus. These sublexical connections between orthographic and phonology are influenced by the regularity/consistency of the diagraph pronunciation and the frequency of the words with which the diagraph appears. Figure 1 presents the relationship between word reading difficulty (based on the d-ELP database), spelling-to-pronunciation transparency (a proxy for word regularity), and word frequency for single-syllable words with the spelling pattern -*eat*. One can see in the figure the well-established word frequency by regularity effect (see Seidenberg et al., 1984) in which the difficulty of high-frequency words is not influenced by regularity (e.g., *eat* vs. *great*), whereas in low-frequency words, regularity of pronunciations affects difficulty (e.g., *cheat* vs. *sweat*).

Evidence for the child-by-word perspective can be further illustrated by examining the relationship between children’s word reading skill and their reading performance on “*ea*-nonwords” (see Steacy et al., 2019; Treiman et al., 2003, 2006). In the Steacy study, the probability of reading a nonword such as *zeam* versus z*ead* (with either the higher-frequency pronunciation of *ea* as/i/vs. the lower-frequency pronunciation of *ea* as/ɛ/) was dependent on a child’s word reading skill and the corpus-based rime support for the vowel pronunciation (-*eam* supports *ea* as/i/whereas -*ead* supports *ea* as/ɛ/). Results suggest that general reading skill at the child level and rime support at the nonword level uniquely facilitate context-dependent vowel pronunciations in our sample of developing readers (i.e., *zeam* read to rhyme with *team* and *zead* to rhyme with *head*). Results were interpreted by Steacy et al. within a statistical learning framework (i.e., a child-by-word framework), supporting a developmental model of word reading in which children are more likely to use an alternative vowel pronunciation in a nonword as they become more proficient readers and as the occurrence of the alternative vowel pronunciation is increasingly supported by the corpus of words experienced. As would be expected in such a model of word reading development, child and corpus attributes work to tune variant vowel pronunciations across individual children and words, with important variance associated with both factors.

## Difficulties predicting word difficulty in developing readers

These relatively simple examples—varied vowel pronunciations of the digraph *ea* in single-syllable words (e.g., *-eat* pronunciations) and nonwords across two fairly frequent rime patterns (*-eam* vs. *-ead*)—illustrate one of the most persistent challenges posed by quasiregular orthographies such as English: accurately predicting word reading difficulty in developing readers while accounting for word characteristics such as frequency, age of acquisition, phonological and morphological complexity, orthographic neighborhood size, and concreteness. Predictive models are essential for quantifying how word-level features interact with child-level knowledge and skill across development—for example, capturing the shift from reliance on simple spelling–sound correspondences to greater use of morphological and syllabic structure during word reading development (see Castles et al., 2018)—while also enabling the testing and refinement of theoretical accounts of lexical development and word recognition. Such models provide a principled framework for examining the child-by-word interactions that underpin variability in reading performance, particularly in populations with reading difficulties, and for understanding how instructional components influence these interactions (e.g., Steacy et al., 2016). Ultimately, understanding these complex interactions between child knowledge—in this case, word reading skill—and word features is critical for developing instructional systems that address the needs of individual students as they learn to read (e.g., Austin et al., 2022; Papalexandratou et al., 2024; U.S. Department of Education, Office of Educational Technology, 2023).

Achieving this goal, however, is complicated by the quasiregular nature of English, which poses significant challenges for generating accurate predictive models across a wide range of words. Existing models tend to be underspecified and often biased by our limited ability to fully capture the quasiregular relations that determine word reading difficulty across words and children (e.g., Lim et al., 2024; Siegelman, Kearns, et al., 2020; Steacy et al., 2022). As a result, the explanatory power of word difficulty predictive models is constrained, reducing their utility for advancing our theoretical understanding of word reading development and providing limited input to instructional design.

**Fig. 2:**
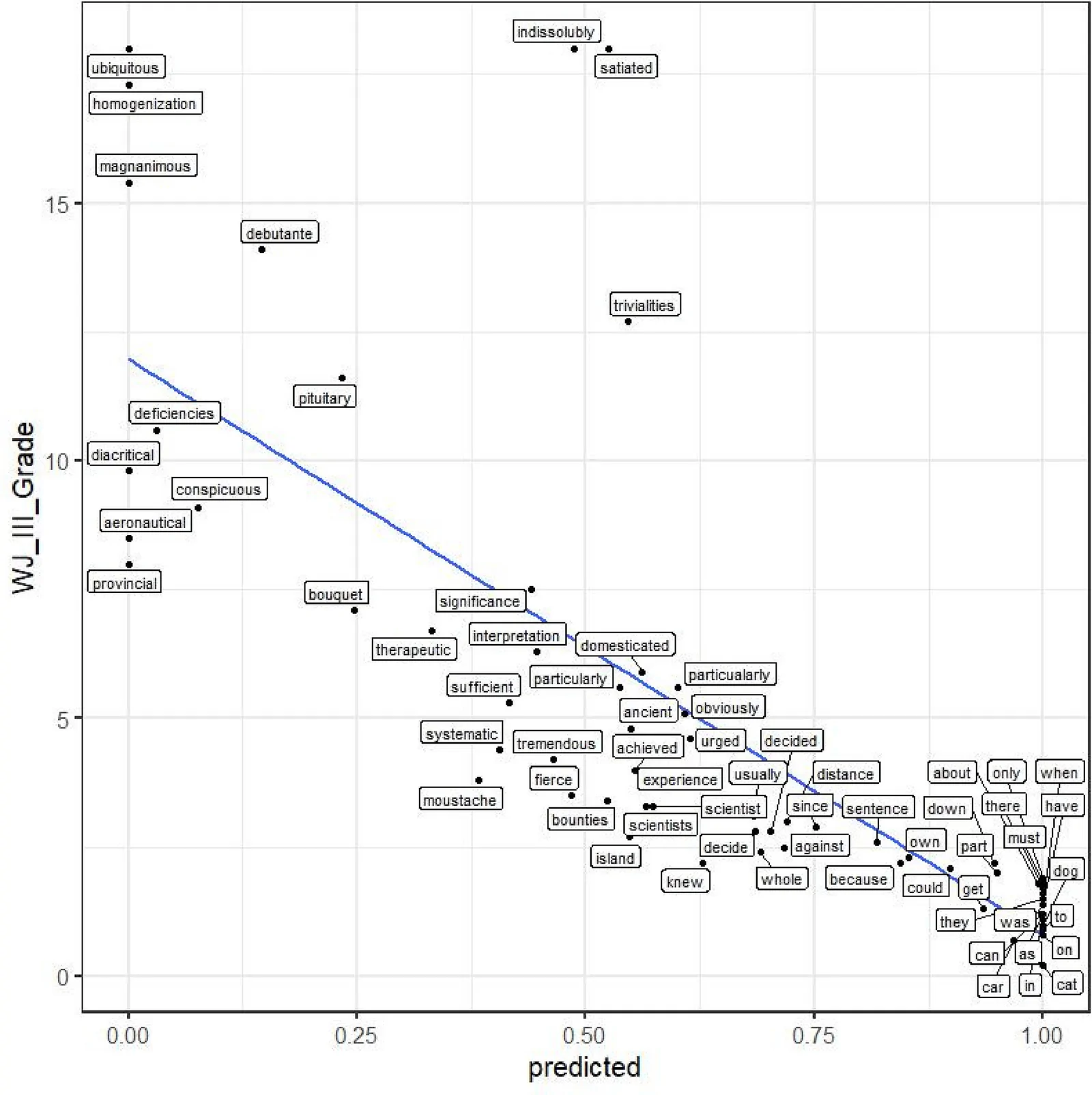
Grade equivalent (GE) scores (*y*-axis) as a function of predicted accuracy (*x*-axis) in WJ3-LWID items. *Note.* Blue trendline is best linear fit.

To illustrate the nature and potential severity of the problem of predicting difficulty across a broad range of word characteristics, we pooled child and word data across multiple studies with the intent of developing a word-level prediction model of accuracy that could be used for predicting item difficulty (grade equivalent [GE]) on the 63 words from the Woodcock-Johnson III Letter–Word Identification (WJ3-LWID; Woodcock et al., 2001) task. Accuracy data from 349 words pooled across five datasets, along with 16 word-level predictors (representing psycholinguistic word properties as well as measures of behavioral performance from the adult ELP), were used as training data to develop a general word difficulty predictive model which was subsequently applied to predict item difficulty on the WJ3-LWID items (for details on the sample and analytic approach, see the supplemental materials). Based on the pooled data, an additive linear regression model predicting (known) accuracy scores was fit from all predictors, with the adjusted-*R*^2^ of this model being 71.7%. This model was applied to the list of 63 WJ3-LWID items (pseudo-*R*^2^ was estimated at 60.4%), and the relation between the predicted accuracy scores and WJ’s GE scores was plotted (Fig. 2). The plot suggests that the predictive model of word difficulty is quite accurate for relatively simple words (i.e., lower right of the figure) but loses precision as words become more complex (i.e., upper left of the figure), as indexed by the distance of the word from the regression line. These results underscore the need for a large-scale behavioral database capturing word reading difficulty across a wide range of word characteristics while sampling the full age range of reading skills in English-speaking children in the early stages of learning to read.

In sum, ample evidence indicates that the quasiregular nature of the English orthography places significant and unique pressures on the development of children’s word reading skills, resulting in pronounced child-by-word effects over the course of development. Understanding the complex interplay between children’s skills and knowledge and the characteristics of words across development remains a critical challenge from both theoretical and instructional perspectives. To help address this need, we introduce the d-ELP—a companion to the English Lexicon Project (Balota et al., 2007)—as an initial resource for examining how English orthography shapes word reading development in children across a large corpus of words. This database provides continuous IRT-based word reading difficulty estimates for 9,961 of the most frequently printed English words for US children in first to fifth grade, along with detailed word-level characteristics and raw trial-level data. In what follows, we detail the methods used to collect the data, analytic framework used to establish a continuous measure of word reading difficulty, descriptive statistics of the database, an explanation of the functionality of the publicly available relational database that provides searchable access to word difficulty ratings and associated word-level statistics for each of the stimuli, and finally a demonstration of the d-ELP’s ability to estimate the impact of general word-level predictors (e.g., frequency, word length, transparency, semantic measures, and orthographic neighborhood density) on word recognition performance in developing readers.

## Method

### Design and procedures

Data collection procedures varied across the project timeline and are described in three formats (Waves 1–3) for the primary data collection, with supplemental archival data included from a prior study. Changes to the data collection procedures across Waves 1–3 were made to increase the probability that children would complete their assigned list of words while also trying to maximize the number of responses per word across the sample. During all aspects of data collection, children worked one-on-one with a trained research assistant in their school, read printed words aloud, and had each response scored as correct or incorrect without performance feedback^2^. For Waves 1–3, children completed only the word reading tasks, with sessions lasting approximately 20 min. In contrast, archival participants completed word reading as part of a larger assessment battery. Across all data collections, children provided verbal assent at the start of the session, and parental consent was obtained beforehand. Data collection procedures were approved by the University Institutional Review Board.

#### Linking items

Two sets of linking items, comprising 99 total words, were used throughout Waves 1–3; the 63 words from the discontinued WJ3-LWID and 36 words from the NIH Toolbox (LaForte et al., 2024). Ceiling rules were only used on the WJ3-LWID, as described by the assessment manual, whereas all other items were only scored if the child attempted that item. For the supplemental archival sample, only the 63 WJ3-LWID words were available as linking items. The primary purpose of these linking items was to place children and words on a common latent reading scale despite the planned-missing design, in which each child read only a subset of the full item pool. Because the same linking words were administered across testing forms and cohorts, they served as common anchors that connected otherwise non-overlapping sets of words and participants. Within the Rasch calibration, performance on the linking items allowed information from individual children to be integrated across forms, enabling the estimation of item difficulty and child reading ability on a common metric. In essence, the linking items provided the psychometric bridge needed to support scale identification and comparability across the multiple forms, cohorts, and waves of data collection. Ceiling rules were applied only to the WJ3-LWID items, following administration procedures described in the assessment manual. All other words were scored only if attempted by the child.

#### Waves 1–3

Children in grades 1–5, 1,388 in total, were presented with a list of printed words (paper or tablet) and asked to read those words aloud. Scores reflected untimed accuracy on each word, with words read correctly scored as a 1 and words read incorrectly scored as a 0. Self-corrections were counted as correct. The assessment ended immediately if a child became visibly frustrated during the word reading task or asked to stop. Assessors received training on identifying pronunciations that may have reflected dialectal or speech-related differences rather than incorrect word identification. When uncertainty arose, assessors documented their observations and audio recordings were reviewed by members of the research team. Final scoring decisions were informed by both the assessor’s notes regarding the child’s speech characteristics and the judgment of experienced investigators. While it is not possible to completely disentangle word recognition difficulty from speech-production factors in an oral reading task, these procedures were implemented to minimize potential scoring errors and ensure consistency in coding.

##### Wave 1

In Wave 1, children were assigned to one of 200 preassembled word lists (40 lists per reading levels 1–5) designed to sample broadly from words at, above, and below their reading level. Before list assignment, children completed the WJ3-LWID task, beginning with the first item and following ceiling rules for stopping and scoring. Raw score (RS) performance on this task determined the grade-level list to which each child was assigned. The following WJ3-LWID RS performance levels were used to assign children to reading level lists: less than 30 RS, Grade 1 list; 30 to 40 RS, Grade 2 list; 41 to 48 RS, Grade 3 list; 49 to 53 RS, Grade list 4; and 54 and greater RS, Grade 5 list. The first set of children (*n* = 67) in Wave 1 received a paper version of the word reading assessment in which the words were printed in columns (three columns of 25 words per page) on physical sheets of paper. Later in Wave 1, students were presented with the same lists in the same order and same number of words and columns per page (again three columns of 25 words per page) but presented on an iPad screen rather than a physical sheet of paper. Students still saw words in a list with columns printed in black words on a white background on an iPad screen. We had hoped to establish ceiling rules for lists that would allow us to assign all words above the ceiling as attempted and incorrect. However, there was enough within- and between-child variability on list reading that ceiling rules were deemed impossible, leading us to only score those items that the child attempted.

##### Wave 2

In Wave 1 we encountered issues of children not finishing their assigned list for various reasons (e.g., inadequate allotment of assessment time or frustration/stamina issues), resulting in some children not attempting all embedded NIH toolbox linking items. Thus, in Wave 2, all 99 linking items (63 WJ3-LWID and 36 NIH Toolbox) were given first so that all children would attempt all linking items. Additionally, in Wave 1, the probability that a word would be attempted by a child decreased the further it was on the list (i.e., it was related by how far a child got on the list), and therefore in Wave 2 the order of words was randomized.

##### Wave 3

In Wave 3 we worked to better match words difficulty to a child’s ability level using initial IRT model results. This was done by first converting their WJ3-LWID performance to an estimated theta based on a preliminary Rasch model. Words with the highest standard error (highest standard error items were chosen because it suggested more data were needed for that item) within a range around the estimated theta were selected for reading lists to increase the number of responses. The range in which words were selected around the estimated theta changed several times during collection of Wave 3 data based on feedback from testers to increase the number of responses from children. The number of pages varied by level, with most having 400 words selected, but at the lower end fewer words were selected so students would be attempting only the most relevant words, with words randomly ordered and split across lists of 50 words. Across Waves 1–3 the maximum number of pages to be attempted was 8.

#### Additional archive data

Data from 519 children who attempted 263 of the same words in a prior data collection were added to the d-ELP sample to increase the overall sample size as well as to increase the number of attempts for these selected words. These children also completed the WJ3-LWID which served as linking items for this sample. This sample was presented with words in a traditional paper format. Children from this data collection have student IDs greater than 99000 in the d-ELP database.

### Participants

Our sampling philosophy for participants was to assess the full range of reading skills across children from the selected grade ranges and allow every child to contribute to the database to the fullest of their ability. Thus, we did not have strict inclusion/exclusion criteria for participation; however, we did ask teachers to identify children within their classroom for which this study was not appropriate, and these children were excluded from assent and assessment. All students with parental consent and child assent were eligible to participate. In rare instances (< 0.2% of potential participants), teachers informed the research team that a student was unlikely to be able to meaningfully participate in the assessment due to significant cognitive, behavioral, or communication challenges. These cases were considered individually when determining participation. Data were collected across schools, both public and private, in five different US states (Connecticut, Florida, Georgia, Maryland, and Pennsylvania). Participants consisted of 1,907 children in grades 1–5^3^; with 331 first graders, 474 second graders, 502 third graders, 360 fourth graders, 217 fifth graders, and 18 with unknown grade level (sample age range, 5.95–13.08 years; *M* = 8.76, *SD* = 1.41; G1 *M* = 6.95, *SD* =.51; G2 *M* = 7.98, *SD* =.51; G3 *M* = 9.01, *SD* =.52; G4 *M* = 10.00, *SD* =.55; G5 *M* = 10.95, *SD* =.44). Of the 1,708 students with completed teacher surveys, 49.6% were female; 36.8% White, 29.8% African American, 14.9% Hispanic, 3.0% Asian, 0.3% Native Hawaiian/Pacific Islander, 0.1% American Indian/Alaskan Native, and 4.6% Multiracial; 5% spoke African American English, 8% were English language learners, and 30% had a disability classification of some type, with the largest percentage having reading difficulties. Standardized WJ3-LWID performance across the sample of participants was *M* = 101.68, *SD* = 15.03, RS min = 39, and RS max = 149.

### Stimuli

Our initial corpus consisted of approximately the 15,000 most frequently printed words in English for school-age children from *The Educator’s Word Frequency Guide* (Zeno et al., 1995). We trimmed the list of words in the following ways: (1) removed all hyphenated words, words with spaces, and words with apostrophes; (2) removed all first names; (3) retained names of major cities, states, and countries; (4) retained names of historical figures (e.g., Einstein); (5) retained names of groups of people (e.g., Egyptians); (6) removed approximately 1,700 simple plurals—addition of “s” with no shifts in orthography or phonology (e.g., bats)—to increase the number of unique words in the database; (7) retained plurals that change orthography or phonology (e.g., cities, changes); (8) added back a set of 75 simple plurals (roughly matched to 75 irregular plurals) to allow for a plurals analysis, and (9) removed words that might be considered objectionable to children, teachers, or parents. This resulted in a list of 10,056 to which we added the linking items. We applied the difficulty prediction model/routine described above and rank-ordered the words from easiest to most difficult. We selected the first 10,000 words from this list (i.e., eliminating 56 words), retaining all linking items, representing the set of words to sample reading accuracy in children grades 1–5.

Graded word lists were initially constructed for Wave 1 using the rank order list with the NIH linking items mixed throughout the list in position of expected difficulty and the WJ3-LWID removed (these items were administered to identify the grade-level list appropriate for each child). Forty lists were built at each reading grade level (1–5) with the idea that lists would sample from a wide range of difficulty while oversampling at the grade level of the list. We anticipated that children at a first grade reading level would be able to read fewer words during an assessment session and so first grade lists were 400 words, whereas second to fifth grade lists contained 500 words. The grade equivalents (GE) of the 63 WJ3-LWID items were used as anchors within the 10,000 words to gauge the general difficulty of words for over sampling at a particular reading grade level.

Sampling for each list varied by reading grade level. Specifically, first grade lists systematically sampled 200 words rank-ordered from 1→1,000 difficulty, 100 from the 1,001→2,000 difficulty, 50 from the 2,001→3,000 difficulty, and 50 from 3,001→5,000 difficulty; second grade lists systematically sampled 100 words from 1→2,000 difficulty, 300 from the 2,001→5,000 difficulty, 50 from the 5,001→6,000 difficulty, and 50 from 6,001→8,000 difficulty; third grade lists systematically sampled 100 words from 1→3,000 difficulty, 300 from the 3,001→6,000 difficulty, 50 from the 6,001→7,000 difficulty, and 50 from 7,001→9,000 difficulty; fourth grade lists systematically sampled 100 words from 1→4,000 difficulty, 300 from the 4,001→7,000 difficulty, 50 from the 7,001→8,000 difficulty, and 50 from 8,001→10,000 difficulty; and fifth grade lists systematically sampled 100 words from 1→4,000 difficulty, 100 from the 4,001→6,000 difficulty, 200 from the 6,001→8,000 difficulty, and 100 from 8,001→10,000 difficulty. Lists within each grade level can be considered roughly equivalent in terms of overall word difficulty with lists becoming systematically more difficult across grade levels.

### Apparatus

A majority of the students completed the word reading assessment on an iPad (whereas the first 67 administrations were presented in a typical paper list format). The iPads were 9th-generation iPads with 10.2-in retina displays with a resolution of 2,160 × 1,620 pixels at 264 pixels per inch. The iPad administration was powered using a Shiny app (Chang et al., 2022) created in R and deployed on the web via shinyapps.io. The administration included a two-screen solution such that the child had an iPad and the tester had an iPad, each with a unique but linked display. The tester would first enter the student ID into both the student and tester iPad. This would either bring up the appropriate list for that child (when using preassigned fixed form lists) during Wave 1 or set the seed for word randomization to be the same on both the student and tester versions during Wave 2–3 (for later versions with randomization to ensure words were randomized yet in the same random order on both the student and tester iPads). The student iPad presented words in black 30-pixel font on a white background arranged in three columns of 25 words each. Words were presented in lowercase letters except when capitalization was required by standard spelling conventions (e.g., proper nouns). The “Word” variable in the database reflects the exact word form presented to participants. The tester iPad looked similar but had a checkbox for scoring next to each word. Testers would select the words which the child read incorrectly (allowing for unchecking for self-corrections, and data were only sent to the database once the tester hit “submit”). In Waves 2 and 3, when a child did not complete the full list, on the page that a child stopped, the tester would click a button indicating “stopped on this page” and then select the last item that the child attempted; the rest of the words (which were not attempted) would be entered into the database as missing. (Wave 1 data underwent a manual data correction procedure to account for where children stopped before this feature was added to the app.) After completion of a page, both the student and the tester would hit the “Next” button at the bottom right of their screen, which would present the next list to be read. After the assessment was complete, the tester hit the “submit” button to send the item response data via the web to secure database storage.

### Analytic approach

#### Creating difficulty estimates

Before moving to IRT modeling, items were explored using classical test theory in order to evaluate which items were appropriate to include in the IRT model. Because of the sampling procedures of items to children, based on expected item difficulty and child ability, proportion correct was not included in this evaluation. The number of correct and incorrect responses were examined along with item correlations with WJ3-LWID total score. A total of 67 items were removed from the subsequent IRT analysis for having negative correlations with the latent word difficulty trait or not having at least two correct and incorrect responses, suggesting the item estimates were not trustworthy. These were removed because items with these characteristics may lead to misleading estimates while not necessarily saying anything about the item itself given our sampling procedures (i.e., it may just be due to insufficient data for that item). This resulted in a total of 9,961 items for model fitting and difficulty estimation.

Next, a Rasch model (with planned missing design) was fit to the child-level data using the TAM package (Robitzsch et al., 2024) in R using marginal maximum likelihood estimation^4^. TAM was selected because it permits estimation of item difficulty in the presence of the planned-missing response structure, in which individual children responded to only a subset of the full item pool. The final dataset used here consisted of 631,322 total responses from 1,907 children across 9,961 words. This package was selected because it offered the ability to model difficulty in the presence of missing data (i.e., not all children saw all words). However, it should be noted that these procedures assume that data are missing completely at random. Although it is true that data were missing because the child did not attempt that word, the specific words that were attempted and not attempted did relate to the child’s ability level in at least two ways: (1) children were assigned lists of words to attempt based partially on likely ability level, and (2) during Wave 1, words were ordered by potential difficulty, with children attempting easier words first, and children often ran out of time/energy before completing the list and tended to attempt fewer words above their ability level. It is possible that there is some relation between whether an item was attempted and a child’s ability level (i.e., difficult words were less likely to be attempted by poor readers). However, given that the linking items attempted by all children span the full range of the item difficulty distribution, we believe this anchored the analysis in a way that allowed item difficulty to be estimated while taking the ability of the students who attempted that item into account. Because the number and reading skill of students who attempted each item differed, it is not the case that estimates for items with more attempts are necessarily more precise, because the precision of the estimate also depends on whether those students represented an appropriate level of ability for that item. For this reason, we avoid using number of attempts in the discussion of precision of individual estimates but still present these data in the relational database. The dataset was collected using a planned-missing design, and thus traditional residual-based global fit indices were not computable. Specifically, the standardized root mean square residuals (SRMR, SRMSR) and Q3-based statistics returned infinite or undefined values, which is expected when item pairs share limited overlap or when some items exhibit near-zero variance among respondents. Despite this, alternative global fit indices indicated acceptable overall model performance. The mean absolute deviation of item covariances (100 × MADCOV = 3.46) suggested that the Rasch model adequately reproduced the observed covariance structure among items. The global homogeneity parameter (GHP = 0.33) was lower than the ideal value of 1.0, indicating that the model underestimates the variability present in the observed data. This underfit likely reflects the sparse response matrix rather than substantive model misspecification. Taken together, the pattern of results suggests that the Rasch model provided a reasonable representation of the data within the constraints of the planned-missing design, though localized dependencies or multidimensionality cannot be ruled out.

Most of the items did not have what is typically considered to be a sufficient sample size for this type of model. The average number of responses per word for nonlinking items was 47.14, with a range of 10–592 responses/word. Given this variability and its effects on difficulty rating, we provide standard errors associated with the difficulty estimate to help users gauge the certainty associated with a given difficulty estimate. It is important to interpret the difficulty estimates in the context of the standard error to note the uncertainty surrounding that estimate. We also provide 95% confidence intervals for the difficulty estimates using this standard error to help the user see the plausible value range for that estimate.

## Creating grade equivalent scores

**Fig. 3:**
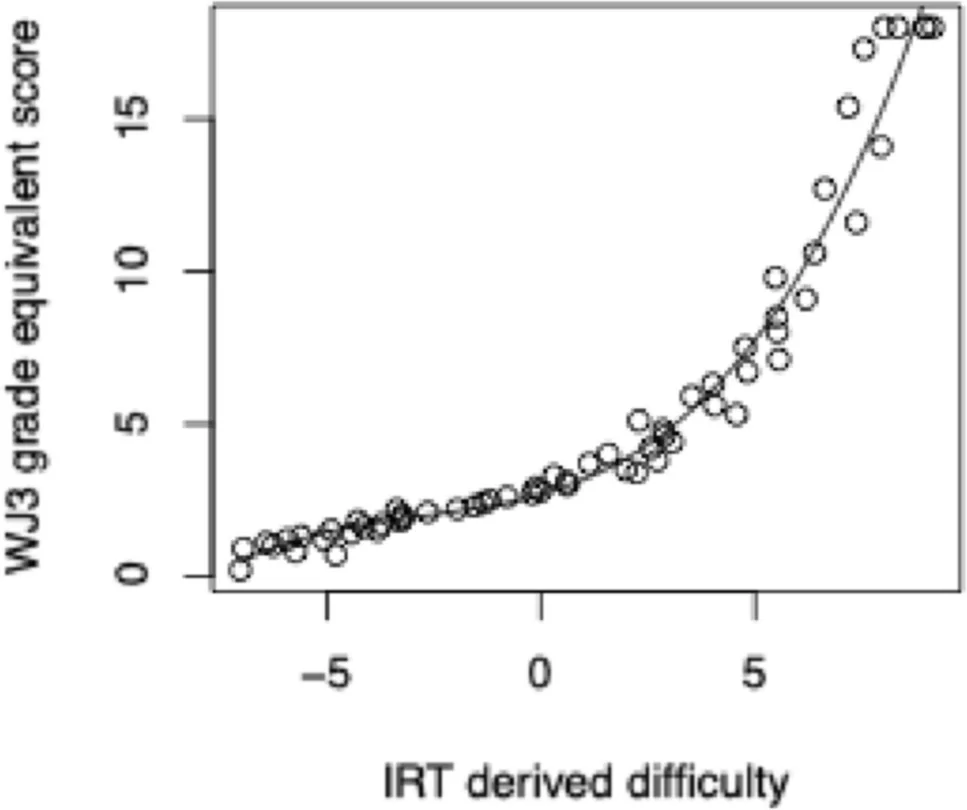
Nonlinear relationship between WJ3-LWID grade equivalent estimates and IRT-derived difficulty for the 63 WJ3-LWID items

Grade equivalent scores were created by first fitting a function for the WJ3-LWID linking items between the IRT-derived difficulty scores and the grade equivalent score from the WJ3-LWID subtest corresponding to the associated raw scores. Plotting the relation between the IRT-derived difficulty and WJ grade equivalent scores revealed a very strong, albeit nonlinear, relation (see Fig. 3). We therefore fitted a function with linear, quadratic, and cubic terms to predict grade equivalence from IRT difficulty, which indeed accounted for 97% of variance in WJ items. The coefficients from this regression were then used to estimate grade equivalent scores from difficulty scores on the full sample of items. This variable is referred to as “grade_eq” in the database.

### Word characteristics

The d-ELP interactive database groups word characteristics into categories—general identifier, word difficulty, occurrence measures, semantics, orthography, phonology, orthography-to-phonology (O2P) and phonology-to-orthography (P2O), and orthography-to-semantics (O2S). We present variables in their current subgroups here to match those in the database. Wherever possible, we retained the names, coding conventions, and values of variables available in existing lexical databases (e.g., CELEX; Baayen et al, 1993; ELP; Balota et al., 2007; SCOPE; Gao et al., 2023) to maximize compatibility with prior research. Because many of these variables were obtained from external sources, they necessarily reflect the coding decisions, conventions, and occasional inconsistencies present in the original databases. Consequently, users should interpret imported variables within the context of their original source and consult the cited references when using variables for which alternative coding conventions exist (e.g., syllable structure, morphological decomposition, or phonological transcription). The d-ELP is intended to provide a comprehensive and extensible resource rather than an authoritative source for every lexical characteristic. Accordingly, the downloadable database preserves the provenance of each variable and enables researchers to substitute or merge alternative lexical variables as appropriate for their research questions. We anticipate that additional variables and updated lexical measures will be incorporated into future releases of the d-ELP. A variable dictionary accompanies the database and provides variable definitions, source citations, and additional documentation for each variable^5^.

### General identifier

***Word_Lowercase.*** The lowercase version of the word, helpful for merging data with varying capitalizations.

***Word.*** Depicts the word (with associated capitalization) as it was presented to the students in the d-ELP word reading assessment.

### Word difficulty

***Difficulty.*** Word reading accuracy item difficulty estimate derived from the Rasch model estimation, with lower values representing easier items and higher values representing more difficult items.

***Standard_Error****.* Standard error associated with the item difficulty derived from the Rasch model.

***Difficulty_Lower****.* Lower bound of the 95% confidence interval around the difficulty estimate. This value was calculated as difficulty − 1.96 × standard_error.

***Difficulty_Upper****.* Upper bound of the 95% confidence interval around the difficulty estimate. This value was calculated as difficulty + 1.96 × standard_error.

***Grade_Eq****.* Grade equivalent of the item difficulty based on the WJ3-LWID grade-based norms. More information on how this was calculated can be found in the “Creating grade equivalent scores” section above.

***N_Attempts.*** Number of children that attempted to read the word.

***N_Correct.*** Number of children that correctly read the word.

***N_Incorrect.*** Number of children that incorrectly read the word.

### Occurrence measures

#### General frequency

***Tasa_SFI.*** Standard Frequency Index taken from Zeno et al. (1995) which is a logarithmic transformation of a word’s frequency in text.

***Tasa_D.*** Dispersion value taken from Zeno et al. (1995) which represents the number of different source documents in which the word appeared.

***Tasa_U.*** Use variable taken from Zeno et al. (1995) which refers to the number of grade-level corpora in which the word occurred. This value ranges from 1 to 4 and indicates the number of grade ranges over which the word was found. The four grade bands were K–2, 3–4, 5–6, and 7–12.

***Tasa_F.*** Frequency variable taken from Zeno et al. (1995) which represents the total raw frequency count of the word across the entire corpus. It indicates how often the word appeared in the 17.25-million-word sample.

***Tasa_Gr1-Tasa_Gr13.*** Grade-level frequency count that indicates how often a word appeared in texts intended for that US school grade level (Zeno et al., 1995).

***Log_Freq_HAL.*** Log10 version of frequency norms based on the Hyperspace Analogue to Language (HAL) corpus (Lund & Burgess, 1996).

***Freq_SUBTLEXUS.*** Log10 version of frequency norms based on the SUBTLEXUS corpus (Brysbaert & New, 2009).

***Sub_Unigram_Freq.*** Frequency of a unigram in the complete subtitle archive of OpenSubtitles.org based on the values reported in subs2vec (van Paridon & Thompson, 2021).

***Wiki_Unigram_Freq.*** Frequency of a unigram in Wikipedia text based on values reported in subs2vec (van Paridon & Thompson, 2021).

***Cyplex_7_9_Raw_Freq.*** Number of times the word appears in books for the 7–9-year age range of the Children and Young People’s Books Lexicon (CYP-LEX) corpus (Korochkina et al., 2024).

***Cyplex_7_9_Zipf_Freq.*** Standardized frequency metric (logarithmic scale) for words appearing in the 7–9-year age range of CYP-LEX (Korochkina et al., 2024).

***Cyplex_7_9_Book_Prop****.* Proportion of books in the 7–9-year age range in the CYP-LEX corpus (Korochkina et al., 2024) in which the word appears.

***Cyplex_10_12_Raw_Freq.*** Number of times the word appears in books for the 10–12-year age range of the CYP-LEX corpus (Korochkina et al., 2024).

***Cyplex_10_12_Zipf_Freq.*** Standardized frequency metric (logarithmic scale) for words appearing in the 10–12-year age range of CYP-LEX (Korochkina et al., 2024).

***Cyplex_10_12_Book_Prop.*** Proportion of books in the 10–12-year age range in the CYP-LEX corpus (Korochkina et al., 2024) in which the word appears.

#### Contextual frequency

***CD_SUBTLEXUS****.* Log10 version of the contextual diversity of a word which refers to the number of passages (documents) in the SUBTLEXUS corpus (Brysbaert & New, 2009) containing a particular word.

### Semantics

#### Familiarity

***Prevalence_Brys.*** Proportion of people who know the word in Brysbaert et al. (2019) in which participants were asked to indicate whether they knew a word in a list of words and nonwords in an online crowdsourcing study. Values were translated to *z*-values based on a cumulative normal distribution. A word known by 2.5% of participants corresponds to a prevalence of −1.96, and a word known by 97.5% of participants corresponds to a prevalence of +1.96.

***Fam_Glasgow.*** A word’s subjective familiarity on a 1 (unfamiliar) to 7 (familiar) scale (Scott et al., 2019).

#### Age of acquisition

***AoA_Kuper****.* Ratings in which participants were asked to enter the age at which they thought they had learned the word (Kuperman et al., 2012). Specifically, it was stated that learning a word means “the age at which you would have understood that word if somebody had used it in front of you, even if you did not use, read, or write it at the time.”

***AoA_LWV****.* Age at which people acquired a word based on a three-choice test administered to participants in grades 4 to 16 (Brysbaert & Biemiller, 2017). This AoA estimate is based on the work of Dale and O’Rourke’s (1981) Living Word Vocabulary database. For the Living Word Vocabulary, Dale and O’Rourke assessed the grade at which children are likely to “know” the meaning of a word by giving students three-alternative multiple-choice options. They assigned each word to the grade level at which 67–80% of the specific word meanings were passed (see Brysbaert & Biemiller, 2017).

#### Part of speech^6^

***DPoS_Brys.*** Dominant grammatical category to which a word is assigned in accordance with its syntactic functions (Brysbaert et al., 2012).

***POS***^7^. A word’s part of speech based on the English Lexicon Project (ELP, Balota et al., 2007).

#### Concreteness and imageability

***Conc_Brys****.* Represents word concreteness, the degree to which the concept can be experienced directly through the senses from a 1 (abstract) to 5 (concrete) scale (Brysbaert & Kuperman, 2014).

***Imageability.*** The ease with which a word can evoke a mental image on a 100 (difficult, low imagery) to 700 (easy, high imagery) scale (Coltheart, 1981).

#### Polysemy

***Nmeanings_Websters.*** Number of meanings based on the Merriam–Websters dictionary (Gao et al., 2023).

#### Affect

***Valence_Warr.*** Pleasantness of a word on a 1 (happy) to 9 (unhappy) scale (Warriner et al., 2013).

***Valence_NRC.*** Word–emotion associations ranging from 0 (negative) to 1 (positive) scale (Mohammad & Turney, 2013).

***Arousal_Warr.*** Intensity of emotion provoked by a word on a 1 (aroused) to 9 (calm) scale (Warriner et al., 2013).

***Arousal_NRC****.* Passiveness or activeness of a word, 0 (low arousal) to 1 (high arousal), calculated via manual annotators who were presented with four words and asked to rank them by activeness and passiveness (Mohammad, 2018).

***Emot_Assoc_Positive.*** Extent to which a word was associated with a positive emotion ranging on 0 (not associated) to 1 (associated) scale (Mohammad & Turney, 2013).

***Emot_Assoc_Joy.*** Extent to which a word was associated with joy ranging on 0 (not associated) to 1 (associated) scale (Mohammad & Turney, 2013).

#### Semantic diversity

***Sem_Diversity.*** Degree to which different contexts associated with a word vary in their meaning (Hoffman et al., 2013).

***Sem_N.*** Number of semantic neighbors based on co-occurrence (Shaoul & Westbury, 2006).

#### Morphology^8^

***NMorph.*** Number of morphemes in a word based on the values in the English Lexicon Project (Balota et al., 2007).

***MorphSp.*** Morphological spelling of a word based on the values in the English Lexicon Project (Balota et al., 2007).

### Orthography

#### Number of letters

***Length.*** Number of letters in a word.

#### Orthographic neighborhood

***Ortho_N.*** Orthographic neighborhood size of a word representing the number of words that can be obtained by changing one letter while preserving the identity and position of the other letters (Balota et al., 2007).

***OLD20.*** Mean orthographic Levenshtein distance of the 20 closest orthographic neighbors of a word (Balota et al., 2007).

***OLD20F.*** Mean Log_Freq_HAL of the closest 20 orthographic neighbors (Balota et al., 2007).

***Orth_Spread.*** Number of letter positions that can be changed to form another word that differs by a single letter (Chee et al., 2020).

#### Bigram frequency

***BG_Sum.*** Summed bigram (two-letter sequence) frequency taken from the English Lexicon Project (Balota et al., 2007).

***BG_Mean.*** Average bigram frequency which is the summed bigram frequency divided by the number of bigrams in a word (Balota et al., 2007).

***BG_Frequency_By_Position.*** Summed bigram frequencies that are sensitive to the positions within words (Balota et al., 2007).

### Phonology

#### Pronunciation

***Pronunciation.*** Machine-readable phonetic alphabet-transcribed pronunciation (SAMPA; Wells, 1997) taken from the ELP (Balota et al., 2007)^9^.

#### Number of phonemes

***NPhon****.* Number of phonemes in a word based on the values in the ELP (Balota et al., 2007).

#### Number of syllables^10^

***NSyll.*** Number of syllables in a word corrected for discrepancies across publicly available databases. ELP values were used when values agreed with those from Chee et al. When the two values disagreed across databases or there were missing data in at least one of the databases, then the value from howmanysyllables.com was used. Number of syllables was resolved in a total of 621 words in which the databases disagreed or there were missing data.

***NSyll_ELP.*** Number of syllables in a word based on the values in the English Lexicon Project (ELP, Balota et al., 2007).

***NSyll_Chee.*** Number of syllables determined algorithmically from phonological transcriptions (Chee et al., 2020).

#### Phonological neighborhood

***Phono_N.*** Number of phonological neighbors or the number of words that can be obtained by changing one phoneme in a word while preserving the identity and position of the other phonemes (Balota et al., 2007).

***PLD20.*** Mean phonological Levenshtein distance of the 20 closest phonological neighbors of a word (Balota et al., 2007).

***PLD20F.*** Mean Log_Freq_HAL of the closest 20 phonological neighbors (Balota et al., 2007).

#### Phonetic features of the first phoneme

***First_Phon_Bilabial.*** Dichotomous variable of whether the first phoneme of a word is a bilabial (Gao et al., 2023; Weide, 2005).

***First_Phon_Labiodental****.* Dichotomous variable of whether the first phoneme of a word is a labiodental (Gao et al., 2023; Weide, 2005).

***First_Phon_Interdental.*** Dichotomous variable of whether the first phoneme of a word is an interdental (Gao et al., 2023; Weide, 2005).

***First_Phon_Alveolar.*** Dichotomous variable of whether the first phoneme of a word is an alveolar (Gao et al., 2023; Weide, 2005).

***First_Phon_Palatal.*** Dichotomous variable of whether the first phoneme of a word is a palatal (Gao et al., 2023; Weide, 2005).

***First_Phon_Velar.*** Dichotomous variable of whether the first phoneme of a word is a velar (Gao et al., 2023; Weide, 2005).

***First_Phon_Glottal.*** Dichotomous variable of whether the first phoneme of a word is a glottal (Gao et al., 2023; Weide, 2005).

***First_Phon_Stop****.* Dichotomous variable of whether the first phoneme of a word is a stop (Gao et al., 2023; Weide, 2005).

***First_Phon_Fricative****.* Dichotomous variable of whether the first phoneme of a word is a fricative (Gao et al., 2023; Weide, 2005).

***First_Phon_Affricate****.* Dichotomous variable of whether the first phoneme of a word is an affricate (Gao et al., 2023; Weide, 2005).

***First_Phon_Nasal.*** Dichotomous variable of whether the first phoneme of a word is a nasal (Gao et al., 2023; Weide, 2005).

***First_Phon_Liquid.*** Dichotomous variable of whether the first phoneme of a word is a liquid (Gao et al., 2023; Weide, 2005).

***First_Phon_Glide.*** Dichotomous variable of whether the first phoneme of a word is a glide (Gao et al., 2023; Weide, 2005).

***First_Phon_Voiced****.* Dichotomous variable of whether the first phoneme of a word is voiced (Gao et al., 2023; Weide, 2005).

### Orthography-to-phonology (O2P) and phonology-to-orthography (P2O)

#### Phonographic neighborhood

***Phonographic_N.*** Number of phonographic neighbors which refers to the number of words that can be obtained by changing one letter and one phoneme while preserving the identity and position of the other letters and phonemes (Balota et al., 2007).

***Phonographic_N_Freq.*** Average Log_Freq_HAL of the phonographic neighborhood of a word (Balota et al., 2007).

#### Spelling-to-pronunciation transparency ratings

***Spell2Pron***^11^. Spelling-to-pronunciation transparency ratings which subjectively measure the ease of arriving at a correct pronunciation for a word by applying typical decoding rules, serving as a global index of regularity between orthography and phonology (Edwards et al., 2024).

#### Consistency

***Consistency_Token_FF_O.*** Spelling-to-sound consistency measure of the word onset calculated as the log frequencies of friends divided by the total log frequencies of friends and enemies (Chee et al., 2020).

***Consistency_Token_FF_R****.* Spelling-to-sound consistency measure of the word rime calculated as the log frequencies of friends divided by the total log frequencies of friends and enemies (Chee et al., 2020).

***Consistency_Token_FB_O.*** Sound-to-spelling consistency measure of the word onset calculated as the log frequencies of friends divided by the total log frequencies of friends and enemies (Chee et al., 2020).

***Consistency_Token_FB_R****.* Sound-to-spelling consistency measure of the word rime calculated as the log frequencies of friends divided by the total log frequencies of friends and enemies (Chee et al., 2020).

### Orthography-to-semantics (O2S)

#### Orthographic–semantic consistency

***Siegelman_OSC****.* Orthographic–semantic consistency represents the level of congruence in meaning between a word and its orthographic neighbors. This value is derived from the mean cosine similarity between the word and its orthographic neighbors in semantic space (Siegelman et al., 2022).

***Marelli_OSC****.* Orthography–semantics consistency measures the degree of semantic relatedness between a word and all words that contain the same letter string as the word (e.g., corn–corner; Marelli & Amenta, 2018).

Descriptive statistics for the word-characteristic variables included in the d-ELP are shown in Table 1. The d-ELP database allows a list of words to be submitted to generate characteristics for the list (see description of the Query function below) or generate a list of words based on a set of specific constraints (see description of the Filter command below). Within both functions the full set of word characteristics/variables can be accessed through drop-down menus. The d-ELP also allows visualization of word sets through a three-dimensional plotting routine (i.e., routine used to generate Fig. 1 above) that allows rotations to better visualize relationships among word characteristics. The database also allows a complete download of the word database along with the full database of deidentified child-level item data (see the description of the Information and Downloads tab below).

**Table 1:** Descriptive data for selected word characteristics in the d-ELP

Word characteristic	*N*	*M*	*SD*	Min	Max
General identifier					
Word	9,961				
Word difficulty					
Difficulty	9,961	0.41	2.02	−7.01	9.14
Standard_Error	9,961	0.49	0.09	0.06	0.85
Difficulty_Lower	9,961	−0.54	2.02	−7.61	8.26
Difficulty_Upper	9,961	1.37	2.04	−6.64	10.01
Grade_Eq	9,961	3.23	1.30	0.47	19.73
N_Attempts	9,961	63.38	179.51	10.00	1,970.00
N_Correct	9,961	42.57	117.80	2.00	1,874.00
N_Incorrect	9,961	20.81	103.76	3.00	1,902.00
Occurrence measures					
Tasa_SFI	9,958	50.47	6.35	19.2	88.38
Tasa_D	9,958	0.71	0.13	0.00	1.00
Tasa_U	9,958	80.40	955.54	0.00	68,006
Tasa_F	9,958	1,393.23	11,782.68	1.00	495,371
Log_Freq_HAL	9,910	8.49	1.70	1.37	16.96
Freq_SUBTLEXUS	9,740	2.48	0.80	0.30	6.31
Cyplex 7–9 RF	9,834	1,021.07	10,123.20	1.00	608,232
Cyplex 10-12_RF	9,930	1,985.09	19,837.75	1.00	1,171,206
CD_SUBTLEXUS	9,740	2.31	.69	0.30	3.92
Semantics					
Prevalence_Brys	8,029	2.30	0.23	0.00	3.00
Fam_Glasgow.	3,200	5.53	0.72	2.00	7.00
AoA_Kuper	6,437	7.77	2.28	2.00	17.00
AoA_LWV	6,163	7.71	2.19	2.00	17.00
Conc_Brys	7,989	3.26	1.05	1.07	5.00
Nmeaning_Websters	7,215	3.09	2.43	1.00	28.00
Valence_Warr	5,858	5.34	1.27	1.40	8.53
Valence_NRC	6,967	0.55	0.22	0.01	1.00
Arousal_Warr	5,858	4.13	0.91	1.60	7.74
Arousal_NRC	6,967	0.48	0.18	0.07	0.97
Emot_Assoc_Pos	5,825	0.19	0.39	0.00	1.00
Emot_Assoc_Joy	5,825	0.06	0,24	0.00	1.00
Sem_Diversity	9,508	1.69	0.31	0.30	2.41
Sem_N	9,726	2,888.79	3,094.36	0.00	9,931.00
NMorph	9,961	1.78	0.72	1.00	5.00
Orthography					
Length	9,961	6.94	2.19	1.00	16.00
Ortho_N	9,910	3.39	5.02	0.00	34.00
OLD20	9,782	2.35	0.82	1.00	7.20
OLD20F	9,782	7.11	0.85	3.87	11.90
Ortho_Spread	9,560	1.12	1.23	0.00	5.00
BG_Sum	9,908	25,553.46	14,692.38	95.00	107,720
BG_Mean	9,908	4,122.96	1,604.26	95.00	14,646
BG_Freq_By_Position	9,908	3,984.26	2,110.07	2.00	13,208
Phonology					
NPhon	9,961	5.76	2.03	1.00	19.00
NSyll	9,961	2.15	0.96	1.00	6.00
Phono_N	9,910	7.40	11.31	0.00	60.00
PLD20	9,782	2.26	0.95	1.00	7.90
PLD20F	9,782	7.28	1.02	3.18	12.01
Ortho-to-Phono (O2P)					
Phonographic_N	9,910	1.99	3.50	0.00	24.00
Phonographic_N_Freq	4,729	6.79	2.10	0.00	15.00
Spell2Pron	9,961	2.21	0.55	1.00	5.27
Consist_Token_FF_O	9,561	0.87	0.15	0.00	1.00
Consist_Token_FF_R	9,561	0.71	0.21	0.00	1.00
Consist_Token_FB_O	9,561	0.83	0.17	0.00	1.00
Consist_Token_FB_R	9,561	0.57	0.24	0.00	1.00
Ortho-to-Semantics (O2S)					
Siegelman_OSC	8,014	0.15	0.20	−0.23	0.98
Marelli_OSC	5,334	0.81	0.27	−0.09	1.00

### Using the interactive database

Users can request access to the interactive database by visiting https://d-elp.org/. Prior to being granted access to the database, we ask users to register and agree to use the data for noncommercial purposes. Once access is granted, the interactive database provides multiple tools and pathways for retrieving and exploring the data. The most basic way is for users to download the full word-level database from the Information and Downloads tab. For users wanting to obtain word characteristics on a specific list of words, the Query tab provides the ability to input words and select which variables to obtain. Alternatively, the Filter tab can be used to find words that meet certain criteria based on their characteristics, filtering the list based on chosen variables of interest. Finally, the raw trial data can be directly downloaded from the Information and Downloads tab.

#### Query

**Fig. 4:**
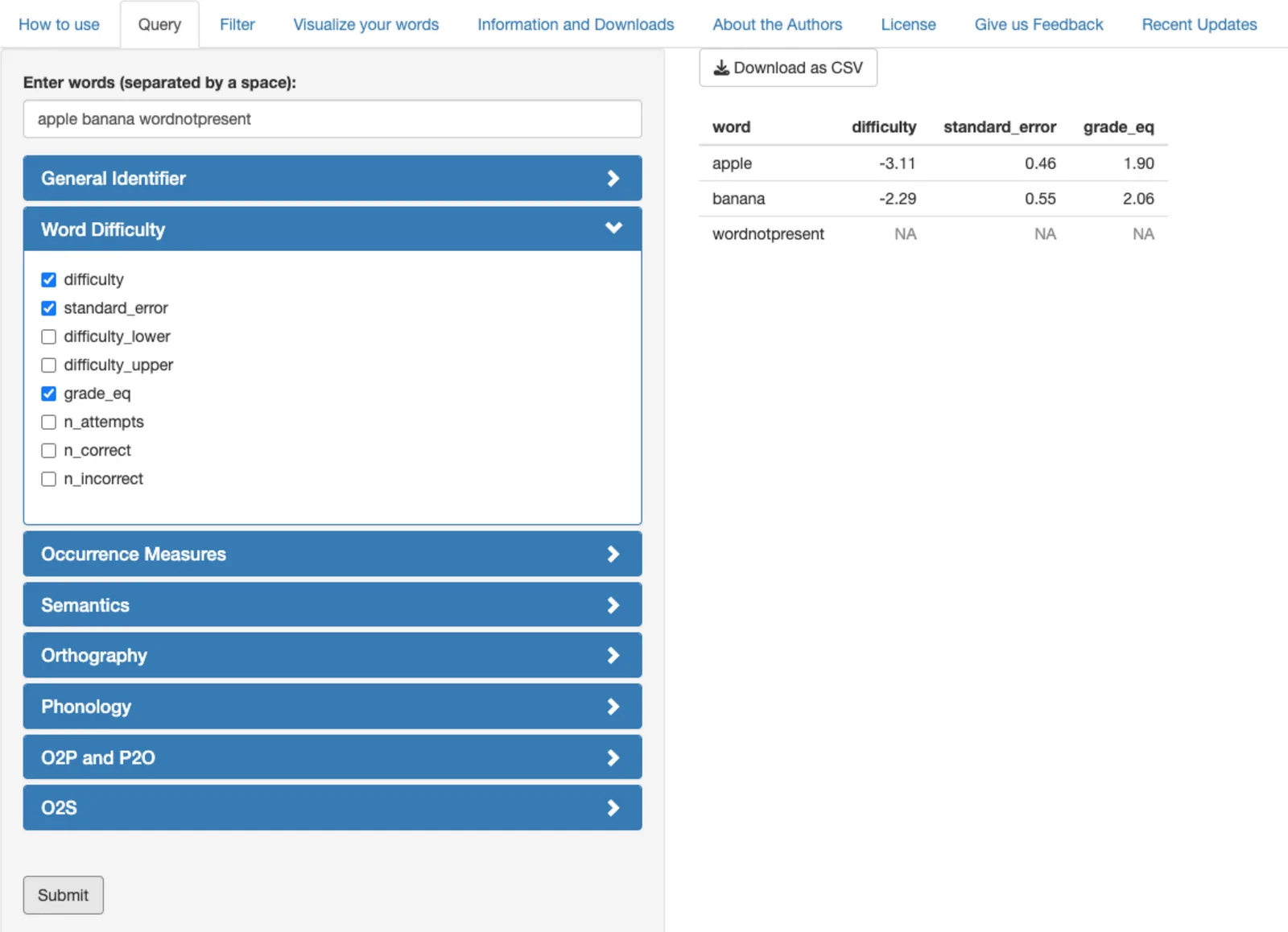
An example of the Query function of the searchable database

The query tab allows users to input (by typing or copying and pasting from Excel) a list of words (separated by spaces) for which they would like associated word characteristics, activated through numerous checkboxes, sorted into expandable accordion groups. Once the user hits submit, the words along with the selected variables will be presented in the table to the right, which can be downloaded using the “Download as CSV” button at the top of the table. Words will appear in rows in the same order that they were entered, and any words that do not appear in the database will still appear in the list but will return “NA” for all of the variables. An example is presented in Fig. 4.

#### Filter

**Fig. 5:**
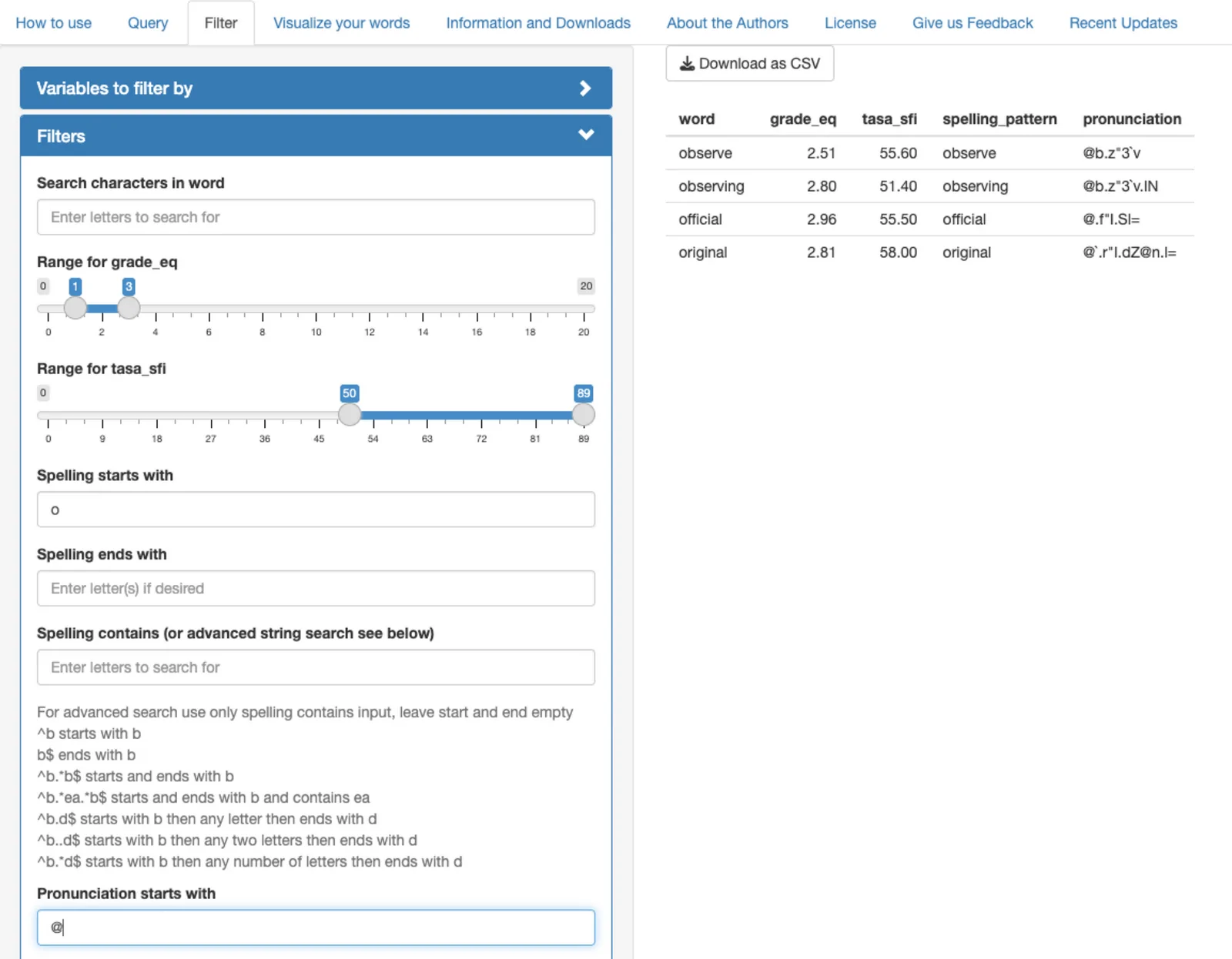
An example of the Filter function of the searchable database

The filter tab is used for finding words in the d-ELP list that meet certain criteria set by the user. First, users select the set of variables that they want to filter. It is important to note that if a word has missing data for any of the selected variables, then it will be removed from the list. Users can then provide specific criteria that words must meet on each of those variables. Numeric variables populate a range in which the upper and lower bounds can be moved to the desired number. Character string variables will populate text boxes to search for characters within that string. Figure 5 shows an example of a user looking for words with difficulty between grades 1 and 3, with standard frequencies above 50, starting with the letter o, and whose pronunciation starts with a schwa.

**Fig. 6:**
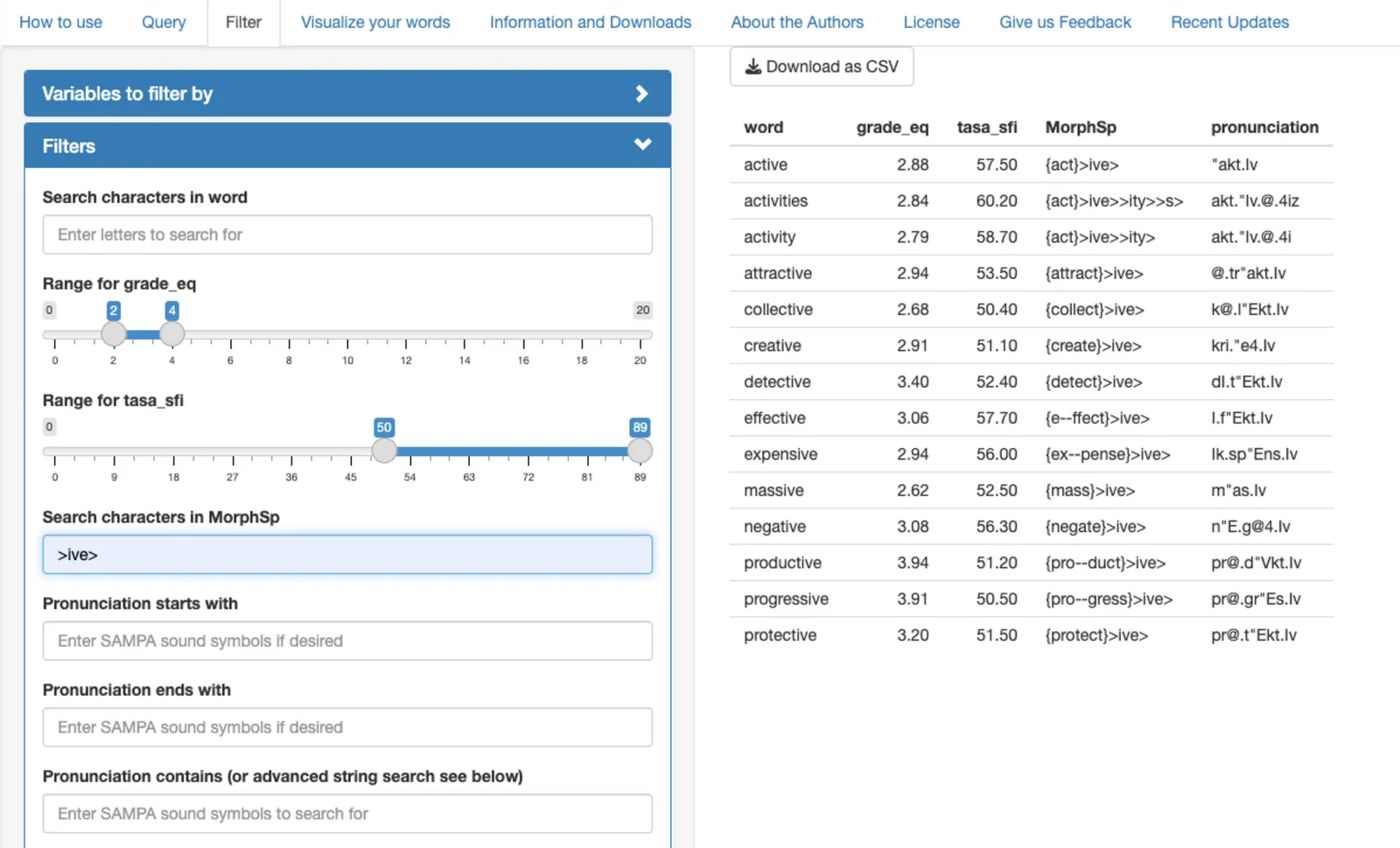
An example of the Filter function of the searchable database using the morphological spelling option

Similarly, Fig. 6 shows a user searching for words with difficulty in the range of grades 2–4, frequencies above 50, and with the “ive” suffix in its morphological spelling. Note that this search is different from the spelling search above, which would be searching for the letters “ive” in the spelling of the word. Here, since we are specifically looking at the morphological spelling, it also returns words like “activity” which does not contain the letter string “ive” but does contain the morpheme.

## Results

### Interpreting difficulty scores

**Fig. 7:**
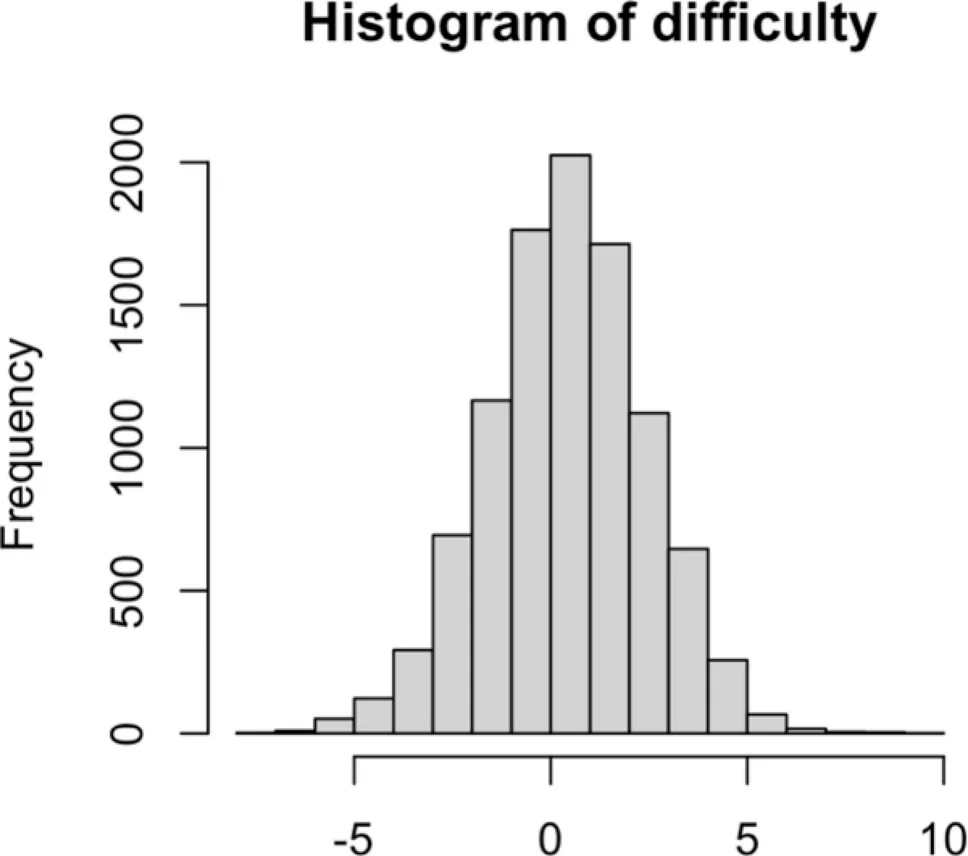
Histogram of difficulty estimates from the Rasch IRT model

Difficulty estimates were derived from the IRT item difficulty parameter in which easier items have lower values and harder items have larger values. Difficulty had a mean of 0.41 and a standard deviation of 2.03, ranging from −7.02 to 9.15 (see Fig. 7). It is important to interpret the difficulty estimates in the context of the associated standard errors to appreciate the uncertainty surrounding individual word difficulty estimates (i.e., the precision of the estimates). It is noteworthy that our average number of responses (47.14 attempts/item for nonlinking items) is in line with other “lexicon projects” across languages (for a summary of average observations per word across studies see Table 1, Stein et al., 2024), with a range of 20–55 average observations per word, with one outlier being the Spanish Lexical Project (SPALEX; Aguasvivas et al., 2018), which had an average of 333 response per word. An important caveat is that the d-ELP database presented here is the only lexicon project to fit an IRT-based difficulty model across a sample of participants differing widely in word reading ability.

As a tangible example of the interpretability of individual difficulty across items, we compare two orthographically related and similarly difficult items “abandon” and “abandoned.” The item “abandon” has a difficulty estimate of 1.02 and “abandoned” has 1.07. While it is reasonable to interpret the difficulty of these words as being close to one another, it is incorrect to conclude that “abandon” is easier than “abandoned.” Looking at the confidence interval, the difficulty of “abandon” is likely to occur anywhere between 0.18 and 1.85, and the difficulty of “abandoned” could be anywhere between 0.08 and 2.06. With these overlapping ranges, we are unable to say for sure that the true difficulty of “abandon” is easier than “abandoned”; however, it would be appropriate to conclude that both items are easier than “abdomen,” which has a difficulty estimate of 4.48 with a plausible value range between 3.32 and 5.63. And so, while caution is warranted when directly comparing difficulty estimates that are close in value, it is also important to note that the Rasch model empirically orders item difficulty by drawing on the full information available in the data, and thus order does matter.

### Interpreting grade equivalent

Grade equivalent (GE) scores are the difficulty scores described above translated to the WJ3-LWID grade equivalent scale norms. These norms were published in 2001 and may not reflect the true performance of students in that grade today. Estimates of difficulty derived from students in grades 1–5 were converted based on WJ3-LWID norm data, with words receiving GE scores outside the grade range of the sample (i.e., 0.4 to 19.7 GE). Similar to difficulty scores, GE scores contain a range of uncertainty around the estimate; words with larger standard errors will have more uncertainty in the grade equivalent scores.

### Example: Established word-level predictors

There are numerous potential uses for the d-ELP database. Here, we present one example illustrating how d-ELP data can be used to evaluate the potential contributions of theoretically important word-level characteristics when included in a model that also contains established word-level characteristics of word difficulty. In defining a set of established word-level characteristics associated with word reading difficulty, we draw on the work of Strain et al. (2002), who demonstrated the independent contributions of various word characteristics (including frequency, word length, transparency, semantic variables, and orthographic neighborhood size) to word recognition performance in adult readers. Our goal here is twofold: (a) to examine whether these word-level characteristics similarly contribute to variation in word difficulty among developing readers, and (b) to demonstrate how failure to account for known sources of lexical variance may result in underspecified models that overestimate the importance of less well-established, though theoretically interesting, predictors.

In this set of regression analyses, we use the following variables as estimates for established word-level characteristics representing each of the following word traits: frequency (Tasa_sfi), word length (Length), transparency (Spell2pron), semantic measures (Conc_Brys and AoA_Kuper), and neighborhood size (Ortho_N). We include two sematic measures as a means of highlighting the influence of word semantic characteristics on word reading difficulty in developing readers (see Kellenberger et al., 2024), variables that are often overlooked as control variables in developmental word reading studies. TASA_SFI was selected for the illustrative analyses because it is derived from educational print materials and was developed to approximate the frequency with which words occur in texts encountered by school-age readers (Zeno et al., 1995). The database also includes several alternative frequency measures (e.g., SUBTLEX-US, OpenSubtitles, HAL, and CYP-LEX) to support investigations using different conceptualizations of lexical exposure. Spell2pron is a consistency rating that asked adults to rate the ease of arriving at a correct pronunciation for a word by applying typical decoding rules. The regression analyses examined both the overall contribution of these word characteristics and the unique variance explained by each predictor after accounting for the remaining variables. Complete data were available for 6,397 words after excluding cases with missing data. Spell2Pron was log-transformed to address skewness and improve linearity with word difficulty. To evaluate potential multicollinearity among predictors, variance inflation factors (VIFs) were examined. All VIF values were low (maximum VIF < 2.0), suggesting that multicollinearity was not a substantial concern in these analyses.

Descriptive statistics and correlations are presented in Table 2. All correlations were statistically significant (*p* <.001). Associations with word reading difficulty ranged from large (AoA_Kuper, Spell2Pron, and Length) to moderate (Ortho_N and TASA_SFI) to small (Conc_Brys). In the initial multiple regression model, all six lexical characteristics were entered simultaneously as predictors of word difficulty. All predictors except concreteness contributed significantly to the model (Table 3, Model 1). Results indicated that words that were longer, less frequent, less transparent, acquired later, and characterized by fewer orthographic neighbors were more difficult to read. Collectively, these predictors accounted for 64.5% of the variance in word reading difficulty across the 6,397 words. We note that, although orthographic neighbors may increase lexical competition in some recognition tasks, larger orthographic neighborhoods have frequently been associated with facilitative effects in word naming, particularly among developing readers, presumably because they provide greater support from familiar orthographic patterns (for details see Andrews, 1997; Carreiras et al., 1997).

To gauge the unique contribution of each variable in predicting word reading difficulty, six additional regression models were run (see Table 3, Models 2–7). This is in keeping with the approach advocated by Balota et al. (2012) of assessing the extent to which a novel predictor accounts for unique variance in megastudies, after controlling for other correlated variables. In these models, the unique *R*^2^ associated with each of the variables (represented by Δ*R*^2^) was estimated by block-entering the other five variables, followed by adding the variable of interest. Overall, Δ*R*^2^ values ranged from.02% to 11.2% of the variance, with the magnitude of unique contributions rank-ordered (from largest to smallest) as follows: age of acquisition, transparency, orthographic neighborhood size, frequency, length, and concreteness. While the results clearly suggest considerable shared variance among the six predictors (67.9% of the explained variance is shared), a sizeable amount of the variance is unique to the various predictors (32.1% of the variance explained is unique). Notably, variables representing age of acquisition, transparency, and orthographic neighborhood size accounted for the largest portion of unique variance but are largely missing as word-level predictors in developmental experiential and individual-differences studies of word reading. This is one example of how the d-ELP can allow researchers to improve their work by providing an evidence-based way to decide which variables to include (here as control variables, but possibly as independent variables as well) in their experimental designs.

## Discussion

The English orthography is best described as quasiregular, consisting of a complex interplay between morphological regularities, syllabic constraints, and numerous sublexical regularities in spelling–sound correspondences. For this reason, English presents a set of unique and significant challenges for children as they work to develop efficient and automatic word reading skills (see Seymour, 2013; Seymour et al., 2003), with individual differences in word reading development representing a complex interaction between the property of words and the skills and knowledge that children bring to the process of learning to read (e.g., Steacy et al., 2022). Teasing out these complex interactions has been difficult due to the complex task of characterizing “word difficulty” from a developmental word reading perspective. The inability to precisely quantify word difficulty empirically across the wide range of words encountered during reading development has also been related to the lack of a large-scale developmental word reading database. To help alleviate these issues, we developed the d-ELP database of word reading performance in a diverse sample of US children. In this first paper, we use these data to derive IRT-based estimates of oral word reading difficulty on a common developmental scale across a broad range of words and to identify several key word-level characteristics that should be considered in future studies of word reading. As with previous “lexicon projects,” we have made our representational database publicly accessible, along with downloadable raw trial data, through the use of an interactive Shiny app. We believe this initial iteration of the d-ELP, and any subsequent versions, will have important educational, psychological, and computational uses going forward that will improve our basic understanding of reading development and practices of improving reading outcomes for children.

## Limitations

Placing the 9,961 most frequently printed English words on a continuous difficulty scale represents a major advancement in promoting the understanding and modeling of word reading difficulty across the early reading developmental span. However, this achievement is best viewed as a foundational step. Because it is impractical to ask children to attempt all 9,961 words, the resulting database necessarily has limitations for modeling child-by-word interactions across both a wide developmental range and a large corpus of words (for details, see Keuleers & Brysbaert, 2010). In addition, the word characteristics contained in the d-ELP were “harvested” from other open-source databases (e.g., CELEX; Baayen et al., 1993; ELP; Balota et al., 2007; SCOPE; Gao et al., 2023) without any type of verification other than spot checks that the merging processes at the item level were accurate. This introduces two immediate problems: (1) there are inaccuracies/inconsistencies in some of the specific word characteristic variables within these mega-databases (e.g., number of morphemes and syllables, stress pattern designation, and age-of-acquisition measures), further complicated by discrepancies occurring across sources, and (2) many of the existing word variables we include in the d-ELP are based on adult corpus (e.g., orthographic–semantic consistency, frequency, semantic diversity) or adult ratings (e.g., spelling-to-pronunciation transparency, familiarity, concreteness). As such, these word characteristics may not necessary be appropriate as measures representing word characteristics in children by systematically overestimating the strengths of word representations as predictors (see Kuperman & Van Dyke, 2013, for the case of age of acquisition). In terms of addressing the first issue, we recommend that when using the d-ELP, some verification be done on variables known to be subjective in nature or to contain inaccuracies. For the second issue we suggest, whenever possible, using word variables based on child corpus or child rating, keeping in mind that there are often not parallel word characteristic variables based on children and adults. Going forward, we acknowledge the need to compute and validate “child-directed measures” for many word characteristic measures used in developmental reading studies; we believe that the d-ELP data would be useful in validating such new quantifications. And finally, subgroup comparisons by ethnicity or language ability were outside the scope of the present study and were not conducted.

Nonetheless, the d-ELP provides a powerful tool for selecting words that match specific developmental periods and linguistic characteristics, thereby facilitating more precise and targeted data collection in which children can reasonably attempt all items. We emphasize that this initial iteration of the d-ELP represents a first step toward a more comprehensive understanding of how word characteristics influence reading difficulty in developing readers. The precision of the current word difficulty estimates is constrained by the number of child-level observations per item, but we aim to establish a mechanism for researchers to contribute additional data to improve item calibration, similar to our integration of archival data in the present study. In this sense, the d-ELP serves as an initial, open-ended framework for estimating and refining word reading difficulty across development.

**Table 2:** Descriptive statistics and correlation coefficients for the lexical predictor analyses (*N* = 6,397)

Variable	*M*	*SD*	Difficulty	Frequency	Length	Trans	Concrete	AoA	Ortho N
Difficulty	0.14	2.11	–						
Frequency	51.13	6.65	−.48	–					
Length	6.62	2.28	.53	−.29	–				
Log Trans	0.74	0.26	.53	−.18	.49	–			
Concrete	3.38	1.07	−.26	−.10	−.29	−.16	–		
AoA	7.75	2.26	.68	−.48	.42	.29	−.37	–	
Ortho N	3.61	5.51	−.49	.24	−.64	−.48	.24	−.35	–

Trans = spelling-to-pronunciation transparency ratings; Concrete = concreteness rating; AoA = age of acquisition; Ortho N = orthographic neighborhood size

All correlations are significant (*p* <.001)

**Table 3:** Hierarchical regression analyses predicting word reading difficulty using the potential control variables (*N* = 6,397) along with estimates of the size of the contribution of each control variable to variance explained (Δ*R*^2^)

Combined model					
	Unstandardized coefficients				
Variable	*B*	*SE*	β	*t*	*p*
Constant	−2.469	0.242		−10.195	<.001
Frequency	−0.053	0.003	−0.166	−18.237	<.001
Length	0.070	0.010	0.076	7.222	<.001
Log Trans	2.529	0.072	0.309	34.962	<.001
Concrete	−0.033	0.017	−0.017	−1.946	0.052
AoA	0.414	0.009	0.443	44.901	<.001
Ortho N	−0.037	0.004	−0.096	−9.509	<.001
**Model 1 – Combined:** *R*^2^ =.645, *F*(6, 6390) = 1,934, *p* <.001 **Model 2 – Unique frequency effects**: Δ*R*^2^ =.019, *F*(1, 6390) = 332.57, *p* <.001 **Model 3 – Unique length effects**: Δ*R*^2^ =.003, *F*(1, 6390) = 52.154, *p* <.001 **Model 4 – Unique transparency effects**: Δ*R*^2^ =.068, *F*(1, 6390) = 1,222.3, *p* <.001 **Model 5 – Unique concreteness effects**: Δ*R*^2^ =.0002, *F*(1, 6390) = 3.787, *p =*.052 **Model 6 – Unique age of acquisition effects**: Δ*R*^2^ =.112, *F*(1, 6390) = 2,016.1, *p* <.001 **Model 7 – Unique ortho neighborhood effects**: Δ*R*^2^ =.005, *F*(1, 6390) = 90.426, *p* <.001					

Model 1 represents the simultaneous addition of predictors into the prediction model; Models 2–7 represent the unique *R*^2^ associated with each of the listed variables when added last to the model in a block entry fashion; Trans = spelling-to-pronunciation transparency ratings; Concrete = concreteness rating; AoA = age of acquisition; Ortho N = orthographic neighborhood size

## Data Availability

The d-ELP relational database is available at: https://qmi-fcrr.shinyapps.io/delp/ and Supplemental materials are available at OSF: https://osf.io/ar9hv/overview?view_only=323ee6d43e204f4781a9802abfbcdf71.

## Footnotes

1 Although lexical decision has served as a primary task in many adult visual word recognition studies and lexicon projects, oral word reading occupies a more central role in English developmental reading research because it directly reflects the acquisition of orthographic-phonological mappings and serves as a primary outcome in educational assessment and intervention research. Accordingly, the present study focused on oral word reading as the basis for estimating item difficulty. We acknowledge that some large-scale adult databases have incorporated both naming and lexical decision measures; however, our objective was to develop a resource that would be maximally informative for understanding and assessing children’s developing word reading skill. Consequently, the d-ELP difficulty estimates should be interpreted as indices of oral word reading difficulty rather than as a broader measure of lexical knowledge or lexical processing.

2 In the case of heteronyms (i.e., words that share the exact same spelling but have different pronunciations and distinct meanings) we accepted either pronunciation as correct.

3 Five students were in grade 6 from the archive data but reading below grade level.

4 A Bayesian-based Rasch model was also fitted to the data, with the correlation between estimates using traditional and Bayesian approaches exceeding.97. We therefore report results from the traditional Rasch approach.

5 Several variables inherited from the ELP (Balota et al., 2007), including those originally derived from CELEX (Baayen et al., 1993), have been noted by previous authors to contain occasional coding inconsistencies or to reflect coding conventions that differ from those adopted in other linguistic resources (e.g., syllable counts, morphological analyses, and some phonological transcriptions; see Chee et al., 2020). To maintain compatibility with the original ELP and facilitate comparison across studies, we retained these variables as originally coded and report their provenance in the variable dictionary. Researchers requiring alternative codings are encouraged to merge them with the downloadable d-ELP database.

6 Part of speech (POS) variable, inherited from the original ELP, includes all parts of speech associated with a word and separates multiple categories using the "|" symbol (e.g., VB|NN for a word that can function as both a verb and a noun). In contrast, DPoS_Brys identifies the dominant part of speech based on the classification provided by Brysbaert and colleagues.

7 Fifty-three words not available in ELP which were coded separately based on the Oxford English Dictionary.

8 Fifty-three words not found in the ELP which were coded by a trained speech-language pathologist.

9 Fifty-three words were coded by a trained speech-language pathologist.

10 We have included three estimates for the number of syllables based on the ELP database (*NSyll_ELP*), Chee et al. database (*NSyll_Chee*), and our attempts to rectify where the two databases do not return the same number of syllables for a word (NSyll). We note that the Chee et al. method for estimating the number of syllables returns more accurate estimates of number of syllables.

11 A total of 807 words on the d-ELP list did not have spelling-to-pronunciation transparency ratings available from Edwards et al. (2024); additional data on these words using the same procedures used in the original study were collected so that all words in the d-ELP could have ratings available. Thus, there are 807 words with spelling-to-pronunciation transparency ratings that have ratings available in d-ELP that were not included in the original set from Edwards et al. (2024).

## Declarations

### Open practices statement

Data and materials are available at https://qmi-fcrr.shinyapps.io/delp/.
